# Impact of age and sex on the efficacy of fremanezumab in patients with difficult-to-treat migraine: results of the randomized, placebo-controlled, phase 3b FOCUS study

**DOI:** 10.1186/s10194-021-01336-1

**Published:** 2021-12-18

**Authors:** Antoinette MaassenVanDenBrink, Gisela M. Terwindt, Joshua M. Cohen, Steve Barash, Verena Ramirez Campos, Maja Galic, Xiaoping Ning, Mikko Kärppä

**Affiliations:** 1 Division of Pharmacology and Vascular Medicine, Department of Internal Medicine, Erasmus University Medical Center Rotterdam, P.O. Box 2040, 3000 CA Rotterdam, The Netherlands; 2 Department of Neurology, Leiden University Medical Center, Leiden, The Netherlands; 3 Teva Pharmaceutical Products R&D, Inc., PA West Chester, USA; 4 Teva Pharmaceuticals Europe B.V., Amsterdam, The Netherlands; 5 Research Unit of Clinical Neuroscience, University of Oulu and Medical Research Center, Oulu University Hospital, Oulu, Finland

**Keywords:** Fremanezumab, Calcitonin gene-related peptide, Age, Sex, Migraine

## Abstract

**Background:**

Migraine prevalence is age and sex dependent, predominating in women in early and middle adulthood; however, migraine also represents a substantial burden for men and adults of all ages. Thus, understanding this burden and the efficacy of migraine preventive medications in both sexes and across age groups is critical. The randomized, placebo-controlled, double-blind, phase 3b FOCUS study demonstrated the safety and efficacy of fremanezumab, a fully humanized monoclonal antibody (IgG2∆a) that selectively targets calcitonin gene-related peptide as a migraine preventive treatment for individuals with migraine and prior inadequate response to 2 to 4 migraine preventive medication classes. Here, we assessed the efficacy of fremanezumab in participants from FOCUS subgrouped by age (18–45 years and > 45 years) and sex.

**Methods:**

In the FOCUS study, eligible participants were randomized (1:1:1) to 12 weeks of double-blind treatment with quarterly fremanezumab, monthly fremanezumab, or matched monthly placebo. In this post hoc analysis, we evaluated changes from baseline in monthly migraine days (primary endpoint of FOCUS) and other secondary and exploratory efficacy outcomes in prespecified age (18–45 and > 45 years) and sex subgroups.

**Results:**

The modified intention-to-treat population (received ≥ 1 dose of study drug and had ≥ 10 days of postbaseline efficacy assessments for the primary endpoint) totaled 837 participants (18–45 years, *n* = 373; > 45 years, *n* = 464; male, *n* = 138; female, *n* = 699). Consistent reductions in monthly average number of migraine days during 12 weeks were observed, regardless of age (18–45 years: quarterly fremanezumab, − 4.1 days; monthly fremanezumab, − 4.7 days; placebo, − 0.9 days; *P* < 0.001; > 45 years: quarterly fremanezumab, − 3.6 days; monthly fremanezumab, − 3.7 days; placebo, − 0.3 days; *P* < 0.001) and sex (male: quarterly fremanezumab, − 4.1 days; monthly fremanezumab, − 4.6 days; placebo, − 0.3 days; *P <* 0.001; female: quarterly fremanezumab, − 3.6 days; monthly fremanezumab, − 3.9 days; placebo, − 0.6 days; *P <* 0.001). Fremanezumab also reduced monthly headache days of at least moderate severity, monthly days of acute medication use, and improved Migraine Disability Assessment scores across subgroups.

**Conclusions:**

These results demonstrate the efficacy of fremanezumab in patients with difficult-to-treat migraine for reducing migraine and headache days, acute medication use, and disability, regardless of age or sex.

**Trial registration:**

ClinicalTrials.gov Identifier NCT03308968 (FOCUS), registered October 13, 2017.

**Supplementary Information:**

The online version contains supplementary material available at 10.1186/s10194-021-01336-1.

## Background

Migraine is a prevalent and burdensome neurological disease that affects > 1 billion people globally [1]. The burden of migraine is heavily age and sex dependent, predominantly affecting women in early and middle adulthood [1]. The American Migraine Prevalence and Prevention Study (AMPP) demonstrated that the prevalence of migraine peaked between 30 and 39 years of age for both men and women; a meta-analysis of 10 European studies demonstrated a similar distribution of migraine prevalence by age group [2, 3].

The AMPP found that the mean prevalence of migraine in women was 17% compared with 6% in men [2, 4], while a meta-analysis of European studies found that the mean prevalence of migraine was 16.6% and 7.5%, respectively [3]. The disparity in prevalence of migraine between women and men is most pronounced among those aged 18 to 29 years [5]. In addition to higher prevalence, migraine in women is associated with higher rates of migraine-related symptoms and higher headache-related disability and impact [5]. The Chronic Migraine Epidemiology and Outcomes (CaMEO) Study found that men may also experience substantial migraine-related disability and are less likely to be diagnosed and treated than women [6]. A consequence of higher prevalence of migraine in women is that there is disproportionate representation of women in studies of migraine preventive treatments; efficacy in males can be difficult to demonstrate due to small subgroup sample sizes. Age and sex differences in migraine prevalence and symptomatology underscore the importance of confirming the efficacy of (preventive) therapies in both men and women, as well as in participants stratified by age. As an example, a recent meta-analysis examined the effect of sex on response to acute treatment with triptans and determined whether these differences were related to pharmacokinetics of triptans in men and women [7]. This study showed sex differences in adverse event frequency, which may be partly because of higher drug exposure in females. Despite higher exposure, women had higher headache recurrence rates, possibly because of longer attack duration related to sex hormonal changes [7].

Fremanezumab, a fully humanized monoclonal antibody (IgG2∆a) that selectively targets calcitonin gene-related peptide (CGRP), has demonstrated safety and efficacy as a migraine preventive treatment for individuals with episodic migraine (EM) or chronic migraine (CM) [8, 9] and is approved for the preventive treatment of migraine in adults [10, 11]. The randomized, double-blind, placebo-controlled, phase 3b FOCUS study demonstrated that fremanezumab is well tolerated and effective in patients with EM or CM and prior inadequate response to 2 to 4 classes of migraine preventive medications [12]. Here, we assessed the impact of fremanezumab on migraine frequency, disability, and acute medication use in individuals from the FOCUS study stratified by age and sex to determine its efficacy in these subgroups.

## Methods

### Study design and participants

The FOCUS study was an international, multicenter, randomized, double-blind, placebo-controlled, parallel-group, phase 3b trial performed at 104 sites; the study design and methods have been described in detail previously [12] and are summarized briefly here. Eligible participants were 18 to 70 years of age and had a diagnosis of migraine with onset at or before 50 years of age. Participants were required to have a history of either EM or CM [13] for ≥ 12 months before screening, in addition to a documented inadequate response within the past 10 years to 2 to 4 of the following pharmacologic classes of migraine preventive medications: beta-blockers (propranolol, metoprolol, atenolol, or bisoprolol), anticonvulsants (topiramate), tricyclic antidepressants (amitriptyline), calcium channel blockers (flunarizine), angiotensin AT_1_ receptor antagonists (candesartan), onabotulinumtoxinA, and valproic acid. Valproic acid was considered as a distinct class from anticonvulsants in general because valproic acid is considered last-line and, in some cases, off-label treatment in some countries. Inadequate response was defined as no clinically meaningful improvement after ≥ 3 months of therapy at a stable dose, as per the treating physician’s judgment; discontinuation because of adverse events that made the medication intolerable; or treatment contraindicated or unsuitable for preventive treatment of migraine for the participant [12]. Exclusion criteria included current use of migraine preventive medications and previous exposure to a monoclonal antibody targeting the CGRP pathway. Full exclusion criteria have been described previously [12].

### Procedures and study assessments

The FOCUS study consisted of a screening visit; a run-in period of 28 days; a 12-week, double-blind, placebo-controlled treatment period; a 12-week, open-label period; and a follow-up visit 6 months after the last dose of study drug. Post hoc subgroup analyses of the 12-week, double-blind, placebo-controlled period are presented here. At baseline, participants were randomized 1:1:1 to receive subcutaneous administration of quarterly fremanezumab, monthly fremanezumab, or matched monthly placebo [12]. For all participants, quarterly fremanezumab consisted of a single 675-mg dose, followed by matched monthly placebo for 2 months. For participants with EM, monthly fremanezumab consisted of 3 monthly 225-mg doses. For participants with CM, monthly fremanezumab consisted of an initial dose of 675 mg, followed by 225 mg for 2 months. For efficacy assessments, participants were asked to record information about headaches during the previous 24-h period using an electronic headache diary device. Subjective ratings of headache severity, total hours of headache for each day, and use of acute migraine medications were also recorded in the electronic diary [12].

### Subgroup analyses

In this post hoc analysis, outcomes were analyzed in participants from the modified intention-to-treat (mITT) population (participants who received ≥ 1 dose of study drug and had ≥ 10 days of postbaseline efficacy assessments for the primary endpoint) in prespecified subgroups according to age and sex. For analyses by age, participants were divided into 2 subgroups: 18 to 45 years of age and > 45 years of age. These age subgroups were prespecified for the analysis of the primary endpoint based on the anticipated distribution of patient ages in the FOCUS study. For analyses by sex, participants were divided into male and female subgroups. The change from baseline in the monthly average number of migraine days during the 12-week double-blind period (primary endpoint for the FOCUS study) and at 4 weeks was assessed. The change from baseline in the monthly average number of headache days of at least moderate severity, the proportion of participants who achieved a ≥ 50% reduction in the monthly average number of migraine days, and the monthly average number of days with acute medication use were also assessed during the 12-week double-blind period. To determine effects of fremanezumab treatment on disability, changes from baseline were assessed by the Migraine Disability Assessment (MIDAS) and the Headache Impact Test-6 (HIT-6) at the end of the 12-week double-blind period.

### Statistical analyses

Changes from baseline in the monthly average number of migraine days, monthly average number of headache days of at least moderate severity, monthly average number of days with acute medication use, MIDAS scores, and HIT-6 scores for fremanezumab treatment groups compared with placebo at all time points were analyzed using a mixed-effects model for repeated measures. Treatment, sex, geographic region, special group of treatment failure (ie, participants who have had documented inadequate response to valproic acid and 2–3 other classes of migraine preventive medications), migraine classification, month, treatment-by-migraine classification interaction, treatment-by-month interaction, and treatment-by-migraine classification-by-month interaction were included as fixed effects; baseline value and years since onset of migraine were included as covariates; and participant was included as a random effect. Comparisons of the proportion of participants who achieved a ≥ 50% reduction in the monthly average number of migraine days in the fremanezumab treatment groups versus placebo were based on a logistic regression model with the following effects: treatment, sex, region, special group of treatment failure, and migraine classification. The least-squares mean change from baseline with standard error is presented for each treatment group. All statistical analyses were generated using SAS software version 9.4 (SAS Institute Inc.).

## Results

### Baseline characteristics

The FOCUS study included 838 patients in the double-blind safety population; 1 patient did not meet the criteria for the mITT population. Thus, this post hoc analysis included 837 participants in the mITT population, of whom 373 (44.6%) were 18 to 45 years of age (quarterly fremanezumab, *n* = 125; monthly fremanezumab, *n* = 128; placebo, *n* = 120) and 464 (55.4%) were > 45 years of age (quarterly fremanezumab, *n* = 151; monthly fremanezumab, *n* = 155; placebo, *n* = 158). There were 138 (16.5%) male participants (quarterly fremanezumab, *n* = 47; monthly fremanezumab, *n* = 45; placebo, *n* = 46) and 699 (83.5%) female participants (quarterly fremanezumab, *n* = 229; monthly fremanezumab, *n* = 238; placebo, *n* = 232). The mean (standard deviation) number of monthly migraine days for participants 18 to 45 years of age at baseline was similar across treatment groups (quarterly fremanezumab, 13.2 [5.68]; monthly fremanezumab, 14.1 [5.75]; placebo, 13.6 [6.34]). Baseline means of monthly migraine days were similar for participants > 45 years of age (quarterly fremanezumab, 14.8 [5.46]; monthly fremanezumab, 14.1 [5.45]; placebo, 15.0 [5.90]). Rates of monthly migraine days at baseline were also similar for male and female participants (male participants: quarterly fremanezumab, 15.1 [5.87]; monthly fremanezumab, 15.9 [5.08]; placebo, 13.8 [6.53]; female participants: quarterly fremanezumab, 13.9 [5.54]; monthly fremanezumab, 13.7 [5.61]; placebo, 14.5 [6.05]; Table 1). The proportion of patients with CM was generally higher in the subgroups of both male and female patients > 45 years of age as compared with the subgroups 18 to 45 years of age (Table 1).

**Table 1:** Baseline Characteristics by Age and Sex: A) Male and B) Female

**A) Male**									
**Age**	**18–45**			**> 45**			**Total**		
	**Placebo** **(*n*** **= 18)**	**Quarterly fremanezumab** **(*n*** **= 18)**	**Monthly fremanezumab** **(*n*** **= 20)**	**Placebo** **(*n*** **= 28)**	**Quarterly fremanezumab** **(*n*** **= 29)**	**Monthly fremanezumab** **(*n*** **= 25)**	**Placebo** **(*n*** **= 46)**	**Quarterly fremanezumab** **(*n*= 47)**	**Monthly fremanezumab** **(*n*= 45)**
Monthly migraine days, mean (SD)	12.6 (5.88)	11.9 (5.16)	15.7 (4.90)	14.6 (6.92)	17.1 (5.47)	16.1 (5.32)	13.8 (6.53)	15.1 (5.87)	15.9 (5.08)
Migraine classification, *n* (%)									
CM	10 (56)	7 (39)	14 (70)	16 (57)	24 (83)	19 (76)	26 (57)	31 (66)	33 (73)
EM	8 (44)	11 (61)	6 (30)	12 (43)	5 (17)	6 (24)	20 (43)	16 (34)	12 (27)
Migraine preventive medications with inadequate response in the past 10 years, *n* (%)									
Beta-blockers	10 (56)	6 (33)	10 (50)	14 (50)	15 (52)	16 (64)	24 (52)	21 (45)	26 (58)
Anticonvulsants	14 (78)	13 (72)	14 (70)	10 (36)	22 (76)	19 (76)	24 (52)	35 (74)	33 (73)
Tricyclics	7 (39)	10 (56)	6 (30)	11 (39)	13 (45)	14 (56)	18 (39)	23 (49)	20 (44)
Flunarizine	4 (22)	5 (28)	5 (25)	5 (18)	0	2 (8)	9 (20)	5 (11)	7 (16)
Candesartan	0	4 (22)	3 (15)	10 (36)	5 (17)	4 (16)	10 (22)	9 (19)	7 (16)
OnabotulinumtoxinA	8 (44)	2 (11)	3 (15)	9 (32)	12 (41)	10 (40)	17 (37)	14 (30)	13 (29)
Valproic acid	5 (28)	5 (28)	10 (50)	8 (29)	12 (41)	8 (32)	13 (28)	17 (36)	18 (40)
Number of previous preventive medication classes with inadequate response, *n* (%)									
2	8 (44)	11 (61)	11 (55)	19 (68)	12 (41)	9 (36)	27 (59)	23 (49)	20 (44)
3	8 (44)	5 (28)	7 (35)	7 (25)	13 (45)	9 (36)	15 (33)	18 (38)	16 (36)
4	2 (11)	2 (11)	2 (10)	2 (7)	4 (14)	7 (28)	4 (9)	6 (13)	9 (20)
**B) Female**									
**Age**	**18–45**			**> 45**			**Total**		
	**Placebo** **(*n*= 103)**	**Quarterly fremanezumab** **(*n*= 107)**	**Monthly fremanezumab** **(*n*** **= 108)**	**Placebo** **(*n*** **= 130)**	**Quarterly fremanezumab** **(*n*** **= 122)**	**Monthly fremanezumab** **(*n* = 130)**	**Placebo** **(*n* = 232)**	**Quarterly fremanezumab** **(*n* = 229)**	**Monthly fremanezumab** **(*n* = 238)**
Monthly migraine days, mean (SD)	13.7 (6.43)	13.4 (5.76)	13.8 (5.86)	15.1 (5.68)	14.2 (5.33)	13.7 (5.41)	14.5 (6.05)	13.9 (5.54)	13.7 (5.61)
Migraine classification, *n* (%)									
CM	57 (55)	59 (55)	64 (59)	84 (65)	79 (65)	76 (58)	141 (61)	138 (60)	140 (59)
EM	45 (44)	48 (45)	44 (41)	46 (35)	43 (35)	54 (42)	91 (39)	91 (40)	98 (77)
Migraine preventive medications with inadequate response in the past 10 years, *n* (%)									
Beta-blockers	55 (53)	64 (60)	59 (55)	81 (62)	61 (50)	80 (62)	136 (59)	125 (55)	139 (58)
Anticonvulsants	77 (75)	85 (79)	82 (76)	85 (65)	93 (76)	101 (78)	162 (70)	178 (78)	183 (77)
Tricyclics	54 (52)	48 (45)	52 (48)	65 (50)	53 (43)	55 (42)	119 (51)	101 (44)	107 (45)
Flunarizine	21 (20)	18 (17)	22 (20)	29 (22)	18 (15)	16 (12)	50 (22)	36 (16)	38 (16)
Candesartan	17 (17)	20 (19)	16 (15)	24 (18)	24 (20)	23 (18)	41 (18)	44 (19)	39 (16)
OnabotulinumtoxinA	25 (24)	31 (29)	25 (23)	34 (26)	30 (25)	33 (25)	59 (25)	61 (27)	58 (24)
Valproic acid	29 (28)	25 (23)	27 (25)	41 (32)	44 (36)	47 (36)	70 (30)	69 (30)	74 (31)
Number of previous preventive medication classes with inadequate response, *n* (%)									
2	49 (48)	49 (46)	53 (49)	66 (51)	68 (56)	60 (46)	115 (50)	117 (51)	113 (47)
3	36 (35)	36 (34)	43 (40)	31 (24)	31 (25)	39 (30)	67 (29)	67 (29)	82 (34)
4	18 (17)	21 (20)	12 (11)	32 (25)	22 (18)	29 (22)	50 (22)	43 (19)	41 (17)

SD, standard deviation

In the FOCUS study, 50% of participants overall had previous inadequate response to 2 migraine preventive medication classes, 32% had inadequate response to 3, and 18% had inadequate response to 4. In this subgroup analysis, male participants from both age groups previously had inadequate response to migraine preventive medication classes in a similar distribution: 30 (54%) of male participants 18 to 45 years of age had inadequate response to 2 preventive medication classes, 20 (36%) had inadequate response to 3, and 6 (11%) had inadequate response to 4; 40 (49%) male participants > 45 years of age had inadequate response to 2 preventive medication classes, 29 (35%) had inadequate response to 3, and 13 (16%) had inadequate response to 4 (Table 1). In female participants, a greater proportion in the older age group had previous inadequate response to 4 preventive medications compared with the younger age group: 151 (47%) female participants 18 to 45 years of age had previous inadequate response to 2 preventive medication classes, 115 (36%) had inadequate response to 3, and 51 (16%) had inadequate response to 4; 194 (51%) female participants > 45 years of age had previous inadequate response to 2 preventive medication classes, 101 (26%) had inadequate response to 3, and 83 (22%) had inadequate response to 4 (Table 1).

### Outcomes in participants subgrouped by age

**Fig. 1:**
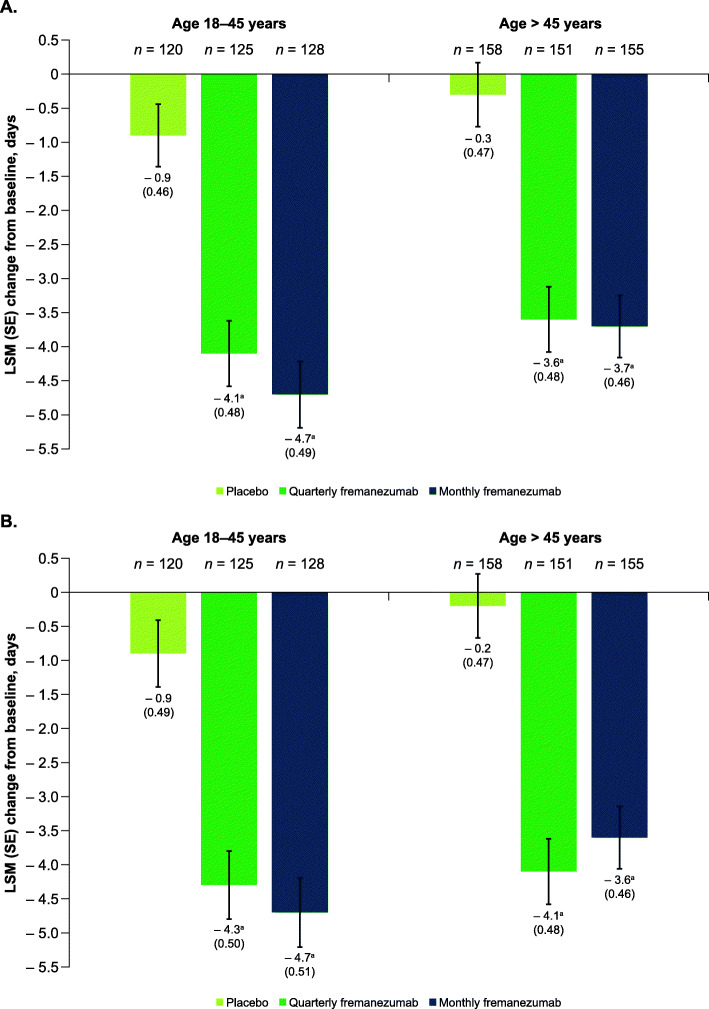
Change in monthly migraine days A) during 12 weeks and B) at 4 weeks by age. LSM, least-squares mean; SE, standard error. ^a^*P* < 0.001 vs placebo

For the primary endpoint of the FOCUS study, both dosing regimens of fremanezumab showed significant reductions in monthly migraine days compared with placebo during 12 weeks of double-blind treatment, regardless of age (Fig. 1A). For participants 18 to 45 years of age, the following reductions in the average monthly number of migraine days were observed: quarterly fremanezumab, − 4.1 days; monthly fremanezumab, − 4.7 days; placebo, − 0.9 days; *P* < 0.001 for both comparisons (Fig. 1A). Although there was a numerical difference in the change from baseline in monthly migraine days between the quarterly and monthly fremanezumab groups for participants 18 to 45 years of age, the difference observed in the change from baseline in monthly migraine days between the 2 dosing groups was not meaningful based on the least-squares mean difference (95% confidence interval [CI]) for monthly fremanezumab versus quarterly fremanezumab (− 0.6 [− 1.54, 0.43]). Similar reductions were observed during 12 weeks of treatment for participants > 45 years of age (quarterly fremanezumab, − 3.6 days; monthly fremanezumab, − 3.7 days; placebo, − 0.3 days; *P* < 0.001 for both comparisons; Fig. 1A). Significant reductions in monthly migraine days were observed as early as 4 weeks after initiation of fremanezumab treatment for participants 18 to 45 years of age (quarterly fremanezumab, − 4.3 days; monthly fremanezumab, − 4.7 days; placebo, − 0.9 days; *P* < 0.001 for both comparisons; Fig. 1B) and > 45 years of age (quarterly fremanezumab, − 4.1 days; monthly fremanezumab, − 3.6 days; placebo, − 0.2 days; *P* < 0.001 for both comparisons; Fig. 1B). Reductions in monthly migraine days were also significant in participants subgrouped by both age and sex (Table 2). There was no significant difference in efficacy between subgroups (male participants versus female participants, *P* = 0.36; male participants 18–45 years of age versus male participants > 45 years of age, *P =* 0.68; female participants 18–45 years of age versus female participants > 45 years of age, *P =* 0.72). When subgroups were evaluated by migraine classification (CM or EM), age, and sex, reductions in monthly migraine days were statistically significant in most subgroups; however, for some subgroups, the small sample size may have limited the ability to detect treatment effects (**Supplementary Table 1**).

**Table 2:** Change in Monthly Migraine Days During 12 Weeks by Age and Sex

Age	18–45			> 45		
	Placebo	Quarterly fremanezumab	Monthly fremanezumab	Placebo	Quarterly fremanezumab	Monthly fremanezumab
Male	(*n* = 18)	(*n* = 18)	(*n* = 20)	(*n* = 28)	(*n* = 29)	(*n* = 25)
LSM (SE) change from baseline, days	− 0.6 (1.16)	− 4.7 (1.41)	− 5.7 (1.26)	− 0.1 (1.07)	− 3.9 (1.05)	− 3.6 (1.14)
*P* value vs placebo		0.010	0.002		0.007	0.006
Female	(*n* = 102)	(*n* = 107)	(*n* = 108)	(*n* = 130)	(*n* = 122)	(*n* = 130)
LSM (SE) change from baseline, days	− 0.5 (0.47)	− 3.5 (0.49)	− 4.0 (0.50)	− 0.7 (0.51)	− 3.9 (0.53)	− 4.1 (0.49)
*P* value vs placebo		< 0.001	< 0.001		< 0.001	< 0.001

LSM, least-squares mean; SE, standard error

**Fig. 2:**
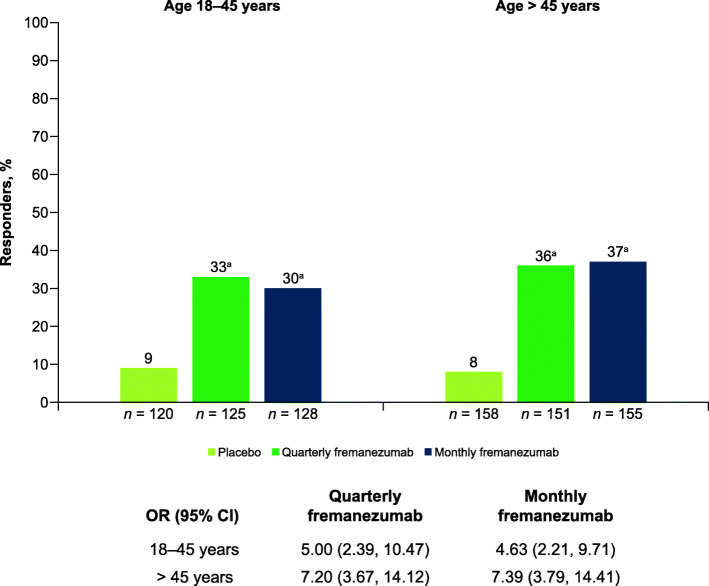
Proportion of patients with ≥ 50% reduction in monthly migraine days from baseline during 12 weeks by age. CI, confidence interval; OR, odds ratio. ^a^*P* < 0.001 vs placebo

Participants from both age groups receiving either dosing regimen of fremanezumab had significant reductions from baseline in monthly headache days of at least moderate severity during 12 weeks of double-blind treatment compared with placebo (18–45 years of age: quarterly fremanezumab, − 3.9 days; monthly fremanezumab, − 4.5 days; placebo, − 0.8 days; *P* < 0.001 for both comparisons; > 45 years of age: quarterly fremanezumab, − 4.1 days; monthly fremanezumab, − 4.2 days; placebo, − 0.5 days; *P* < 0.001 for both comparisons; **Supplementary Fig. 1**). The proportion of participants 18 to 45 years of age with a ≥ 50% reduction in monthly migraine days from baseline during 12 weeks of treatment was significantly greater for both fremanezumab treatment groups compared with placebo (quarterly fremanezumab, 33%; monthly fremanezumab, 30%; placebo, 9%; *P* < 0.001 for both comparisons; Fig. 2). Similar results were observed in participants > 45 years of age (quarterly fremanezumab, 36%; monthly fremanezumab, 37%; placebo, 8%; *P* < 0.001 for both comparisons; Fig. 2).

**Fig. 3:**
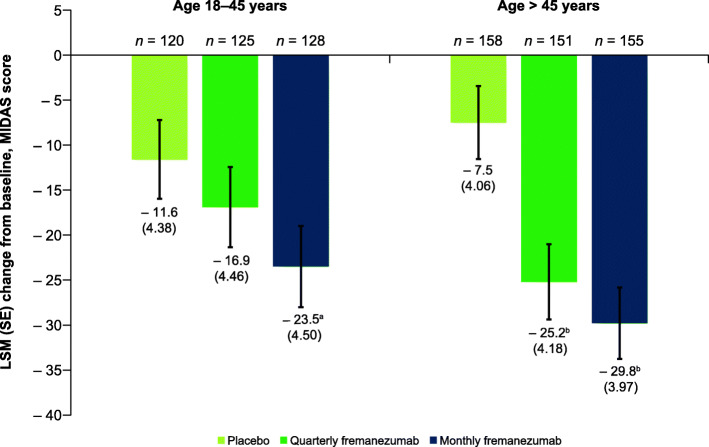
Change in MIDAS scores at 12 weeks by age. MIDAS, Migraine Disability Assessment; LSM, least-squares mean; SE, standard error. ^a^*P* = 0.021 vs placebo. ^b^*P* < 0.001 vs placebo

Participants from both age groups receiving fremanezumab had significant reductions in monthly days of acute medication use to treat migraine symptoms (**Supplementary Fig. 2**). For participants 18 to 45 years of age, the changes from baseline in days of acute medication use during 12 weeks of double-blind treatment were: quarterly fremanezumab, − 4.0 days; monthly fremanezumab, − 4.1 days; placebo, − 1.1 days; *P* < 0.001 for both comparisons (**Supplementary Fig. 2**). Participants > 45 years of age had similar reductions: quarterly fremanezumab, − 3.6 days; monthly fremanezumab, − 3.9 days; placebo, − 0.1 days; *P* < 0.001 for both comparisons (**Supplementary Fig. 2**). Fremanezumab treatment was also associated with decreases in MIDAS scores after 12 weeks, indicating reductions in migraine-associated disability; however, this effect was more robust in older participants. For participants 18 to 45 years of age, MIDAS scores were significantly reduced from baseline in the monthly fremanezumab group (− 23.5, *P* = 0.021) but not the quarterly fremanezumab group (− 16.9, *P =* 0.304) compared with placebo (− 11.6; Fig. 3). For participants > 45 years of age, significant reductions in MIDAS scores were observed with both fremanezumab treatment groups compared with placebo: quarterly fremanezumab, − 25.2; monthly fremanezumab, − 29.8; placebo, − 7.5; *P* < 0.001 for both comparisons (Fig. 3). Both dosing regimens of fremanezumab treatment led to significant reductions from baseline in HIT-6 scores compared with placebo, regardless of age group (**Supplementary Fig. 3**). For participants 18 to 45 years of age, reductions from baseline in HIT-6 scores during 12 weeks of treatment were: quarterly fremanezumab, − 5.3, *P* = 0.008; monthly fremanezumab, − 6.1, *P* < 0.001; placebo, − 3.1 (**Supplementary Fig. 3**). For participants > 45 years of age, reductions from baseline in HIT-6 scores during 12 weeks of treatment were: quarterly fremanezumab, − 5.9; monthly fremanezumab, − 6.7; placebo, − 2.1; *P* < 0.001 for both comparisons (**Supplementary Fig. 3**).

### Outcomes in participants subgrouped by sex

**Fig. 4:**
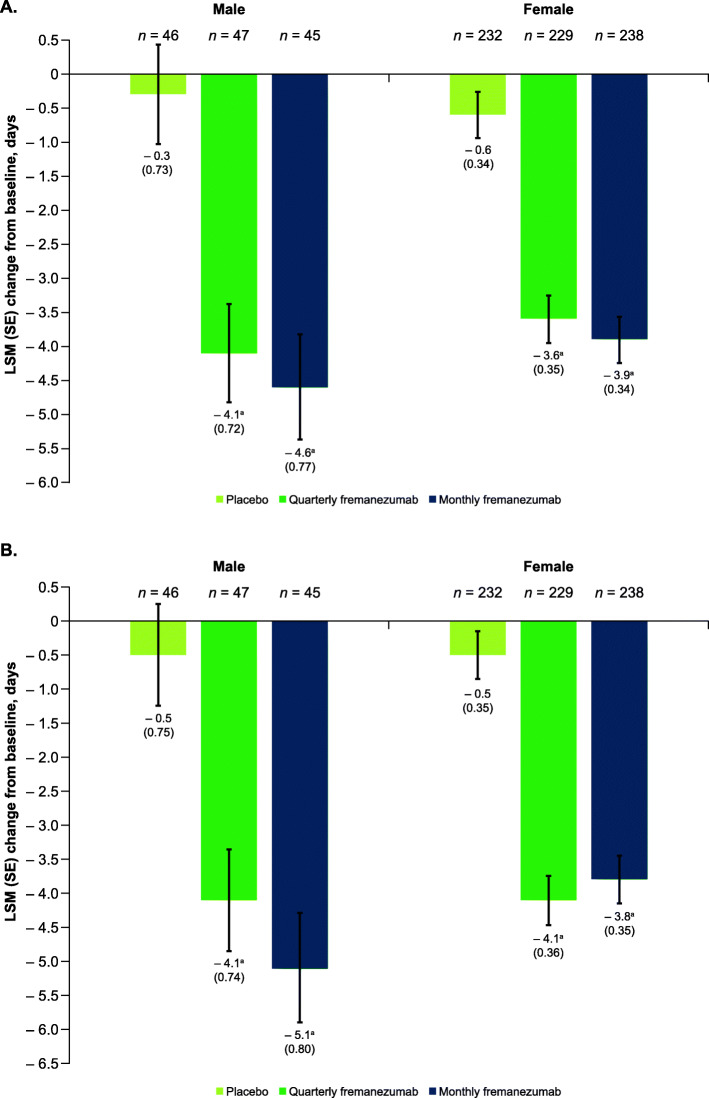
Change in monthly migraine days A) during 12 weeks and B) at 4 weeks by sex. LSM, least-squares mean; SE, standard error. ^a^*P* < 0.001 vs placebo

**Fig. 5:**
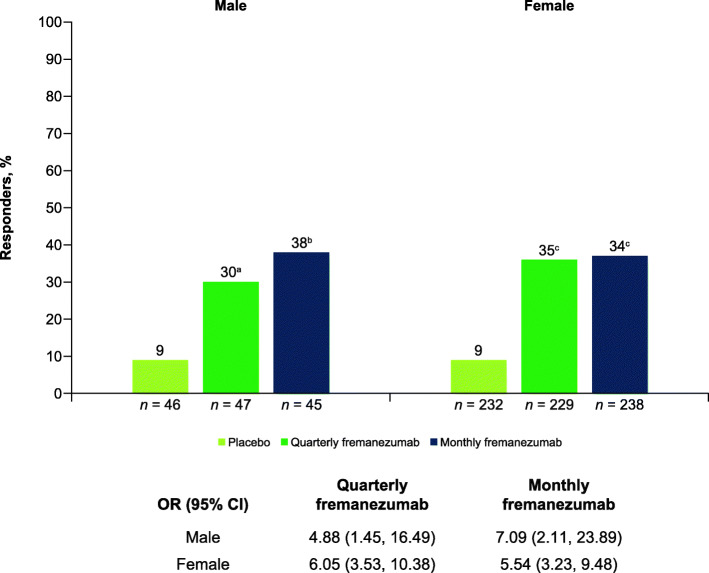
Proportion of patients with ≥ 50% reduction in monthly migraine days from baseline during 12 weeks by sex. OR, odds ratio; CI, confidence interval. ^a^*P* = 0.011 vs placebo. ^b^*P* = 0.002 vs placebo. ^c^*P* < 0.001 vs placebo

Despite the smaller sample size for male participants, significant reductions in monthly migraine days and monthly headache days of at least moderate severity were observed with both fremanezumab dosing regimens compared with placebo, regardless of sex. In male participants, for the primary endpoint, the monthly average number of migraine days was significantly reduced from baseline in both fremanezumab treatment groups compared with placebo during 12 weeks of double-blind treatment (quarterly fremanezumab, − 4.1 days; monthly fremanezumab, − 4.6 days; placebo, − 0.3 days; *P <* 0.001 for both comparisons; Fig. 4A) and as early as 4 weeks after initiating study treatment (quarterly fremanezumab, − 4.1 days; monthly fremanezumab, − 5.1 days; placebo, − 0.5 days; *P <* 0.001 for both comparisons; Fig. 4B). Similar results were observed in female participants. For the primary endpoint, during 12 weeks of treatment, both dosing regimens resulted in significant reductions in monthly migraine days (quarterly fremanezumab, − 3.6 days; monthly fremanezumab, − 3.9 days; placebo, − 0.6 days; *P <* 0.001 for both comparisons; Fig. 4A). Significant reductions in monthly migraine days were observed as early as 4 weeks after treatment initiation in female participants (quarterly fremanezumab, − 4.1 days; monthly fremanezumab, − 3.8 days; placebo, − 0.5 days; *P <* 0.001 for both comparisons; Fig. 4B). Both male and female participants also had significant reductions in monthly headache days of at least moderate severity during 12 weeks of treatment with both fremanezumab dosing regimens compared with placebo (male participants: quarterly fremanezumab, − 4.2 days; monthly fremanezumab, − 4.5 days; placebo, − 0.5 days; *P <* 0.001 for both comparisons; female participants: quarterly fremanezumab, − 3.9 days; monthly fremanezumab, − 4.2 days; placebo, − 0.7 days; *P <* 0.001 for both comparisons; **Supplementary Fig. 4**). The proportion of male participants with a ≥ 50% reduction in monthly migraine days from baseline during 12 weeks of treatment was significantly greater with both fremanezumab dosing regimens compared with placebo (quarterly fremanezumab, 30%, *P =* 0.011; monthly fremanezumab, 38%, *P* = 0.002; placebo, 9%; Fig. 5). Similar results were observed in female participants (quarterly fremanezumab, 35%; monthly fremanezumab, 34%; placebo, 9%; *P* < 0.001 for both comparisons; Fig. 5).

**Fig. 6:**
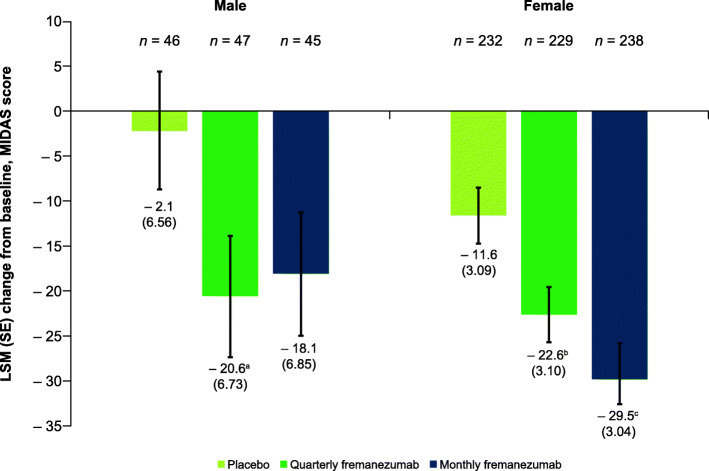
Change in MIDAS scores at 12 weeks by sex. MIDAS, Migraine Disability Assessment; LSM, least-squares mean; SE, standard error. ^a^*P* = 0.023 vs placebo. ^b^*P* = 0.004 vs placebo. ^c^*P* < 0.001 vs placebo

Both male and female participants had significant reductions in monthly days of acute medication use during 12 weeks of fremanezumab treatment compared with placebo (male participants: quarterly fremanezumab, − 4.0 days; monthly fremanezumab, − 4.7 days; placebo, − 0.1 days; *P <* 0.001 for both comparisons; female participants: quarterly fremanezumab, − 3.6 days; monthly fremanezumab, − 3.9 days; placebo, − 0.7 days; *P <* 0.001 for both comparisons; **Supplementary Fig. 5**). Male participants had significant reductions in MIDAS scores after 12 weeks of treatment with both fremanezumab dosing regimens, indicating improvements in migraine-associated disability (quarterly fremanezumab, − 20.6, *P* = 0.023; monthly fremanezumab, − 18.1, *P* = 0.052; placebo, − 2.1; Fig. 6). Similar results were observed in female participants (quarterly fremanezumab, − 22.6, *P* = 0.004; monthly fremanezumab, − 29.5, *P* < 0.001; placebo, − 11.6; Fig. 6). For male participants, reductions in HIT-6 scores from baseline in participants treated with fremanezumab were not statistically significant compared with the placebo group (quarterly fremanezumab, − 4.1, *P* = 0.677; monthly fremanezumab, − 4.7, *P* = 0.401; placebo, − 3.5; **Supplementary Fig. 6**). In female participants, HIT-6 scores were significantly reduced from baseline after 12 weeks of fremanezumab treatment compared with placebo (quarterly fremanezumab, − 5.8; monthly fremanezumab, − 6.8; placebo, − 2.5; *P* < 0.001 for both comparisons; **Supplementary Fig. 6**).

## Discussion

This post hoc analysis of the FOCUS study provides evidence for the efficacy of quarterly and monthly fremanezumab as a preventive migraine therapy for individuals with difficult-to-treat EM or CM with prior inadequate response to multiple other preventive medication classes, regardless of age or sex. We observed significant reductions in monthly migraine days and monthly headache days of at least moderate severity, as well as increased proportions of participants with a clinically meaningful ≥ 50% reduction in monthly migraine days with fremanezumab treatment compared with placebo in participants 18 to 45 years of age or > 45 years of age. Similar results were observed in both male and female participants. The changes from baseline in migraine frequency measures in these subgroup analyses were similar to those observed in the overall study [12]. Acute medication use was also significantly reduced in fremanezumab treatment groups compared with placebo, regardless of age or sex.

These data also suggest a benefit of fremanezumab for reducing participants’ disability, regardless of age or sex. Fremanezumab treatment reduced migraine-associated disability compared with placebo for participants in both age groups and of both sexes, as measured by MIDAS scores, although the difference did not reach statistical significance for participants 18 to 45 years of age receiving quarterly fremanezumab. Based on HIT-6 scores, headache-related disability was significantly reduced with fremanezumab treatment compared with placebo for participants in both age groups and for female participants but not for male participants. The relative sample size of male participants in the FOCUS study was small (only 16% of the overall study population), which may explain the lack of statistical significance for HIT-6 score reduction in male participants. The small proportion of male patients in the current study was in line with that in similar studies of migraine preventive treatments (15%–19%) [14–17]. Thus, finding statistically significant outcomes across a range of efficacy measures in the male population in this study, despite the traditionally low enrollment of male participants, demonstrates that fremanezumab is able to effectively prevent migraine in males.

Data describing age- or sex-specific efficacy of other CGRP pathway−targeting monoclonal antibodies is limited: a post hoc subgroup analysis of the phase 3 STRIVE trial assessed the efficacy and safety of erenumab in women with menstrual migraine, finding that women ≤ 50 years of age with a history of menstrual migraine had reductions in monthly migraine days consistent with the overall study population [18]. To our knowledge, similar subgroup analyses have not been published for galcanezumab or eptinezumab. However, sex differences in the role of CGRP within the trigeminovascular system, as well as in clearance rates for some migraine preventive treatments, have been investigated in animal and preclinical studies. For example, activation of the CGRP system has been shown to fluctuate based on the rat estrous and human menstrual cycles, indicating the impact of sex hormones on trigeminal nociceptive pathways critical to migraine [19]. Fluctuations in female sex hormones, specifically estrogen, modulate CGRP and migraine prevalence [19]. Perimenopause is associated with an increased prevalence of migraine. Furthermore, the burden of migraine and headache frequency often rise during midlife for women, which may be attributed to the menopausal transition [20]. Sex differences in clearance rates and bioavailability of non–migraine-specific preventive medications, such as beta-blockers and calcium channel blockers, have been identified [21]. In the setting of this evidence, determining that fremanezumab is effective regardless of age or sex helps broaden the understanding of the impact of CGRP in migraine pathophysiology between sexes and over age-related periods of hormonal transition within women.

One limitation of this study is that, as a post hoc analysis, there is potential for Type I error due to multiple comparison bias. However, all data points for these analyses were collected a priori, the subgroups were prespecified, and the effect sizes observed for age and sex subgroups of participants were similar to those observed in the overall study population [12]*.* In addition, this study is not powered to detect a difference in the magnitude of the improvements in efficacy outcomes with fremanezumab treatment between age and sex subgroups. The FOCUS study enrolled a broad population of patients with CM and EM and inadequate response to 2 to 4 prior classes of migraine preventive treatment; male patients were not specifically recruited for that study. Detecting small differences would require very large sample sizes that were beyond the scope of this study. Future meta-analyses could test this hypothesis, underscoring the importance of reporting study outcomes in male and female participants separately. Future studies investigating the impact of the interaction between age and sex on the efficacy of fremanezumab should also be considered (eg, the efficacy of fremanezumab during and outside of the menstruation period, in women taking estroprogestinic therapy). Although the subgroup of participants 18 to 45 years of age may generally be considered premenopausal, while the subgroup > 45 years of age likely includes a mix of pre-, peri-, and postmenopausal women, further research is needed to fully assess the efficacy of fremanezumab in women based on menopausal status. Nevertheless, the improvements observed with fremanezumab treatment were similar across age and sex subgroups both in terms of absolute values and effect sizes.

These data provide valuable insights for male participants with migraine, supporting the efficacy of fremanezumab for migraine preventive therapy within the male subgroup. While higher rates of migraine-related symptoms and greater disability have been observed in females with migraine compared to males, migraine is still burdensome for males [6]. Self-reported data indicate that males with migraine are less likely to be on preventive therapy [5], suggesting that males with migraine may be undertreated or underdiagnosed. This represents an opportunity for improvement in the treatment of male patients with migraine.

## Conclusions

This post hoc analysis of the phase 3b FOCUS study demonstrated the efficacy of fremanezumab for migraine preventive treatment in patients with migraine and prior inadequate response to migraine preventive therapies, regardless of age or sex. Despite the relatively small number of male patients recruited, the primary efficacy analysis and several key secondary analyses still reached statistical significance. A larger sample size would have been helpful to confirm the trend for the efficacy analysis for MIDAS and HIT-6, which are not as sensitive as the primary efficacy measure. Nevertheless, these results support the use of fremanezumab as a treatment option for both male and female adults aged 18 to 70 years with difficult-to-treat migraine.

## Supplementary Information

Additional file 1: **Supplementary Table 1.** Change in Monthly Migraine Days From Baseline During 12 Weeks by Migraine Classification, Age, and Sex.

Additional file 2: **Supplementary Fig. 1.** Change in monthly average number of headache days of at least moderate severity during 12 weeks by age. LSM, least-squares mean; SE, standard error. ^a^*P* < 0.001 vs placebo. **Supplementary Fig. 2.** Change in monthly days with acute medication use during 12 weeks by age. LSM, least-squares mean; SE, standard error. ^a^*P* < 0.001 vs placebo. **Supplementary Fig. 3.** Change in HIT-6 scores at 12 weeks by age. HIT-6, Headache Impact Test-6; LSM, least-squares mean; SE, standard error. ^a^*P* = 0.008 vs placebo. ^b^*P* < 0.001 vs placebo. **Supplementary Fig. 4.** Change in monthly average number of headache days of at least moderate severity during 12 weeks by sex. LSM, least-squares mean; SE, standard error. ^a^*P* < 0.001 vs placebo. **Supplementary Fig. 5.** Change in monthly days with acute medication use during 12 weeks by sex. LSM, least-squares mean; SE, standard error. ^a^*P* < 0.001 vs placebo. **Supplementary Fig. 6.** Change in HIT-6 scores at 12 weeks by sex. HIT-6, Headache Impact Test-6; LSM, least-squares mean; SE, standard error. ^a^*P* < 0.001 vs placebo.

## Data Availability

The datasets analyzed during the current study are available from the corresponding author on reasonable request.

## Abbreviations

AMPP: American Migraine Prevalence and Prevention Study
CaMEO: Chronic Migraine Epidemiology and Outcomes Study
CGRP: calcitonin gene-related peptide
CM: chronic migraine
EM: episodic migraine
HIT-6: Headache Impact Test-6
LSM: least-squares mean
MIDAS: Migraine Disability Assessment
mITT: modified intention-to-treat
SD: standard deviation
SE: standard error
