# Supplementary material for: Impact of age and sex on the efficacy of fremanezumab in patients with difficult-to-treat migraine: results of the randomized, placebo-controlled, phase 3b FOCUS study
Source: J Headache Pain. 2021 Dec 18;22(1):152. doi: 10.1186/s10194-021-01336-1 (PMC8903667; doi:10.1186/s10194-021-01336-1)
Supplement: Supplementary file 1 — Supplementary Table 1. Change in Monthly Migraine Days From Baseline During 12 Weeks by Migraine Classification, Age, and Sex. [file 10194_2021_1336_MOESM1_ESM.docx]

**Supplementary Table**

**Supplementary Table 1** Change in Monthly Migraine Days from Baseline During 12 Weeks by Migraine Classification, Age, and Sex

|  | **18-45** | | | **>45** | | |
| --- | --- | --- | --- | --- | --- | --- |
|  | **Placebo** | **Quarterly fremanezumab** | **Monthly fremanezumab** | **Placebo** | **Quarterly fremanezumab** | **Monthly fremanezumab** |
| *CM* |  |  |  |  |  |  |
| Male | (*n* = 10) | (*n* = 7) | (*n* = 14) | (*n* = 16) | (*n* = 24) | (*n* = 19) |
| LSM (SE) change from baseline, days | 0.5 (1.80) | – 4.4 (2.41) | – 6.2 (1.66) | 0.7 (1.27) | – 4.1 (1.01) | – 4.2 (1.20) |
| *P* value vs placebo |  | 0.074 | 0.004 |  | 0.001 | 0.002 |
| Female | (*n* = 57) | (*n* = 59) | (*n* = 64) | (*n* = 84) | (*n* = 79) | (*n* = 76) |
| LSM (SE) change from baseline, days | – 0.2 (0.69) | – 3.9 (0.74) | – 4.8 (0.70) | – 1.9 (0.70) | – 4.2 (0.67) | – 4.4 (0.68) |
| *P* value vs placebo |  | < 0.001 | < 0.001 |  | 0.003 | 0.002 |
| *EM* |  |  |  |  |  |  |
| Male | (*n* = 8) | (*n* = 11) | (*n* = 6) | (*n* = 12) | (*n* = 5) | (*n* = 6) |
| LSM (SE) change from baseline, days | – 2.4 (1.25) | – 5.0 (1.44) | – 6.6 (1.54) | – 2.9 (1.63) | – 3.0 (1.65) | – 4.2 (1.60) |
| *P* value vs placebo |  | 0.089 | 0.039 |  | 0.972 | 0.428 |
| Female | (*n* = 45) | (*n* = 48) | (*n* = 44) | (*n* = 46) | (*n* = 43) | (*n* = 54) |
| LSM (SE) change from baseline, days | – 1.0 (0.62) | – 3.5 (0.60) | – 3.3 (0.65) | 0.6 (0.66) | – 3.3 (0.73) | – 3.6 (0.61) |
| *P* value vs placebo |  | < 0.001 | 0.002 |  | < 0.001 | < 0.001 |

CM, chronic migraine; LSM, least-squares mean; SE, standard error; EM, episodic migraine.
