# Supplementary material for: Impact of age and sex on the efficacy of fremanezumab in patients with difficult-to-treat migraine: results of the randomized, placebo-controlled, phase 3b FOCUS study
Source: J Headache Pain. 2021 Dec 18;22(1):152. doi: 10.1186/s10194-021-01336-1 (PMC8903667; doi:10.1186/s10194-021-01336-1)
Supplement: Supplementary file 2 — Supplementary Fig. 1. Change in monthly average number of headache days of at least moderate severity during 12 weeks by age. LSM, least-squares mean; SE, standard error. aP < 0.001 vs placebo. Supplementary Fig. 2. Change in monthly days with acute medication use during 12 weeks by age. LSM, least-squares mean; SE, standard error. aP < 0.001 vs placebo. Supplementary Fig. 3. Change in HIT-6 scores at 12 weeks by age. HIT-6, Headache Impact Test-6; LSM, least-squares mean; SE, standard error. aP = 0.008 vs placebo. bP < 0.001 vs placebo. Supplementary Fig. 4. Change in monthly average number of headache days of at least moderate severity during 12 weeks by sex. LSM, least-squares mean; SE, standard error. aP < 0.001 vs placebo. Supplementary Fig. 5. Change in monthly days with acute medication use during 12 weeks by sex. LSM, least-squares mean; SE, standard error. aP < 0.001 vs placebo. Supplementary Fig. 6. Change in HIT-6 scores at 12 weeks by sex. HIT-6, Headache Impact Test-6; LSM, least-squares mean; SE, standard error. aP < 0.001 vs placebo. [file 10194_2021_1336_MOESM2_ESM.docx]

**Supplementary Material**

**Supplementary Fig. 1** Change in monthly average number of headache days of at least moderate severity during 12 weeks by age. **
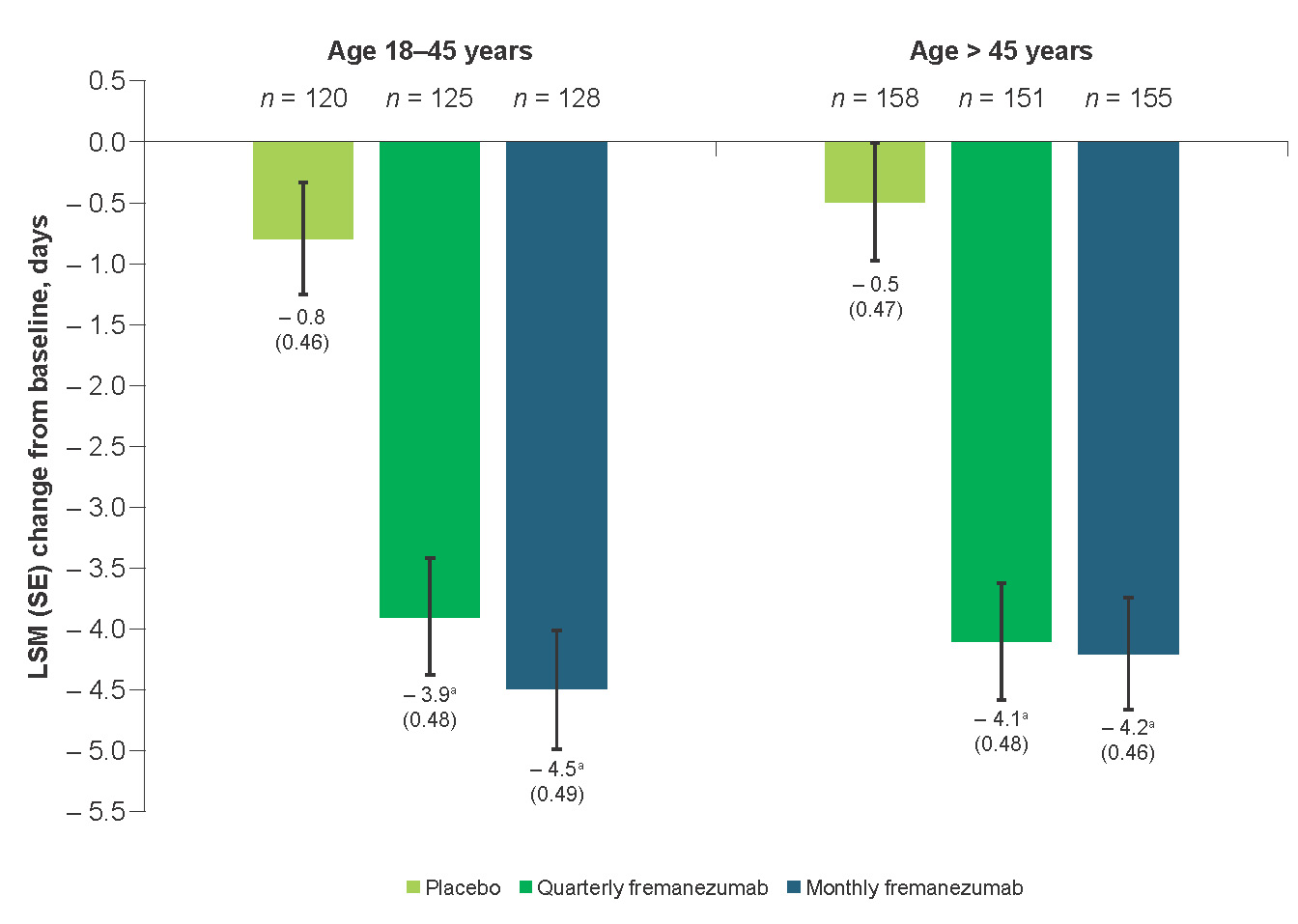
**

LSM, least-squares mean; SE, standard error.

^a^*P* <0.001 vs placebo.

**Supplementary Fig. 2** Change in monthly days with acute medication use during 12 weeks by age.


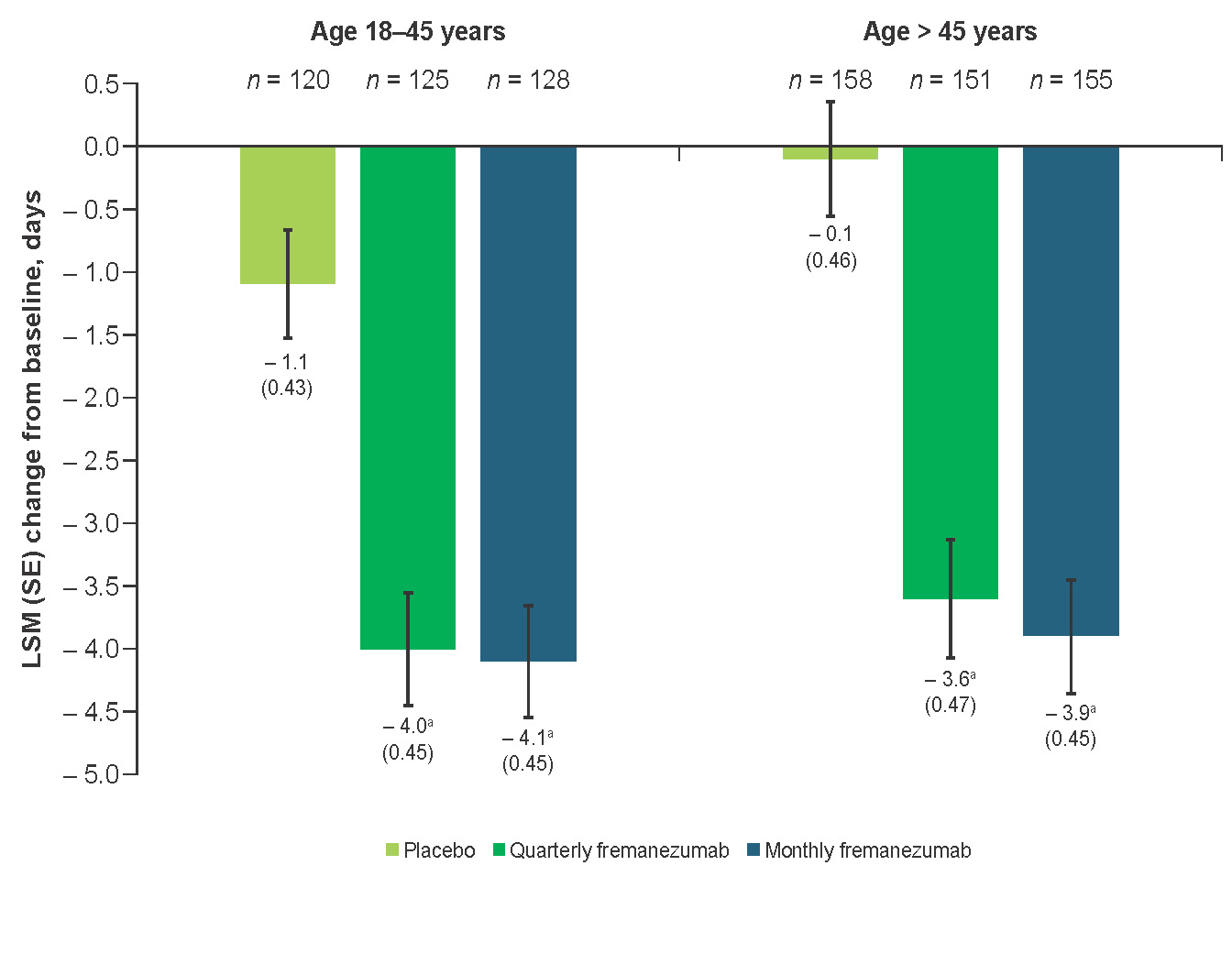


LSM, least-squares mean; SE, standard error.

^a^*P* <0.001 vs placebo.

**Supplementary Fig. 3.** Change in HIT-6 scores at 12 weeks by age.


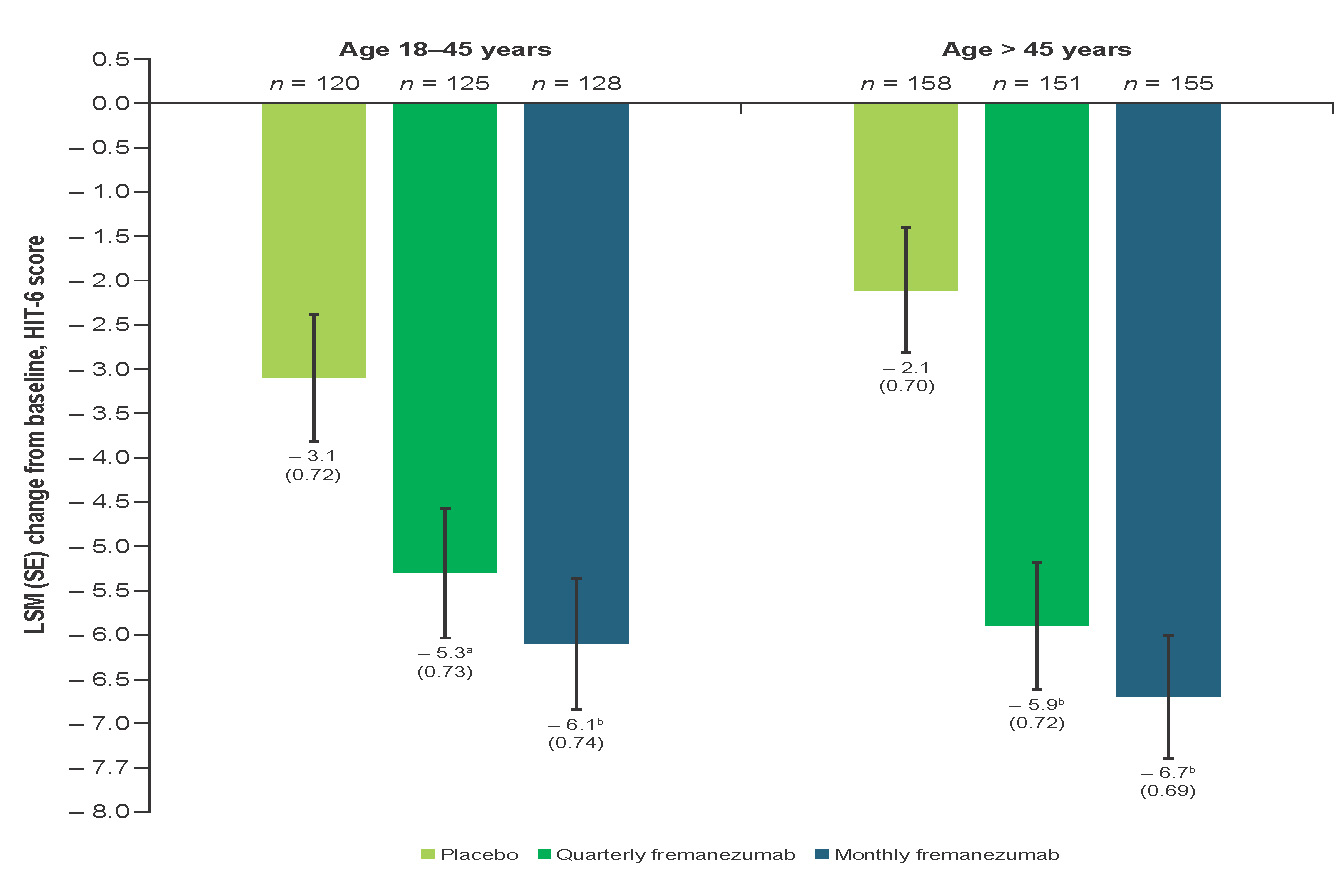


HIT-6, Headache Impact Test-6; LSM, least-squares mean; SE, standard error.

^a^*P* = 0.008 vs placebo.

^b^*P* <0.001 vs placebo.

**Supplementary Fig. 4.** Change in monthly average number of headache days of at least moderate severity during 12 weeks by sex.


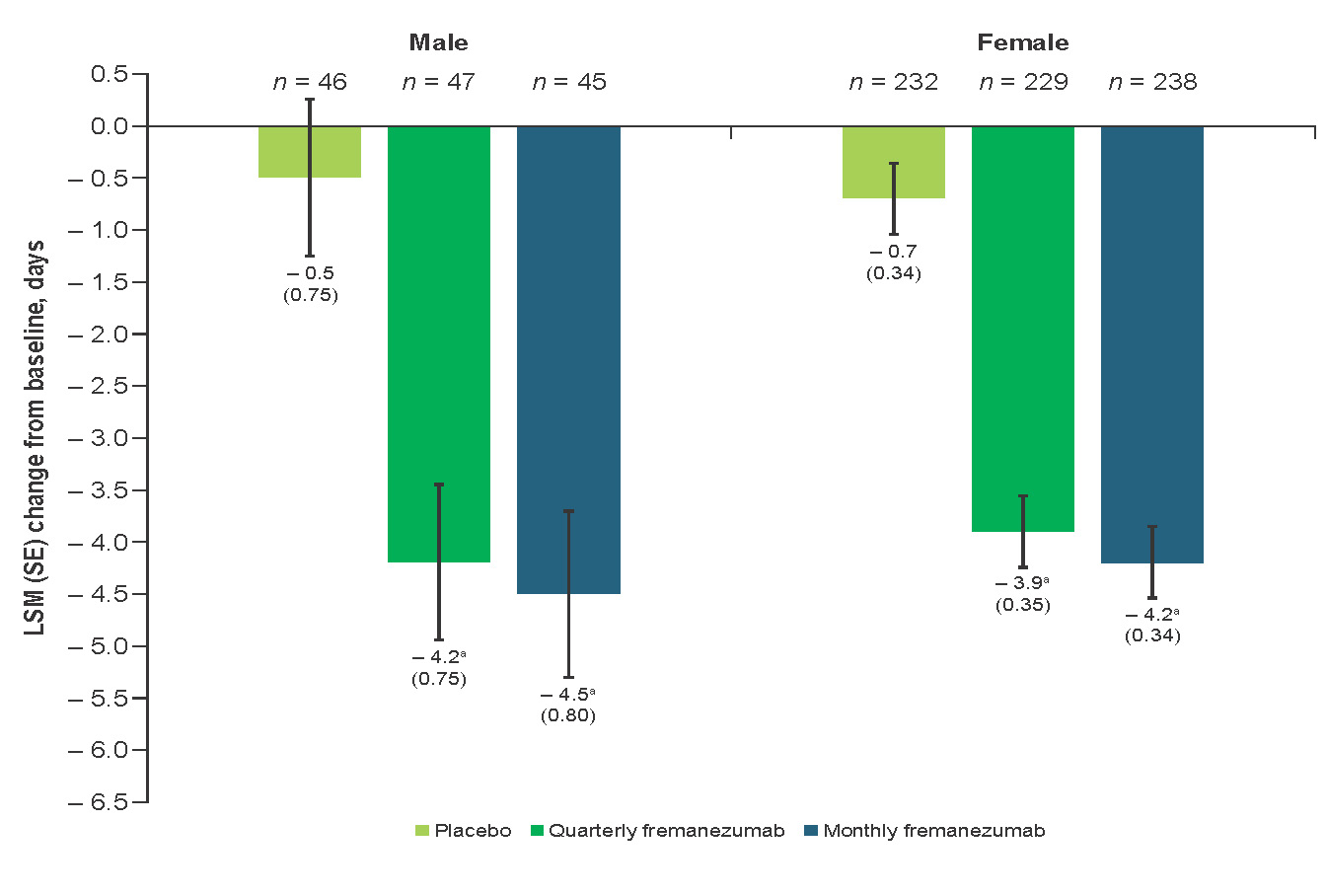


LSM, least-squares mean; SE, standard error.

^a^*P* <0.001 vs placebo.

**Supplementary Fig. 5.** Change in monthly days with acute medication use during 12 weeks by sex.

**
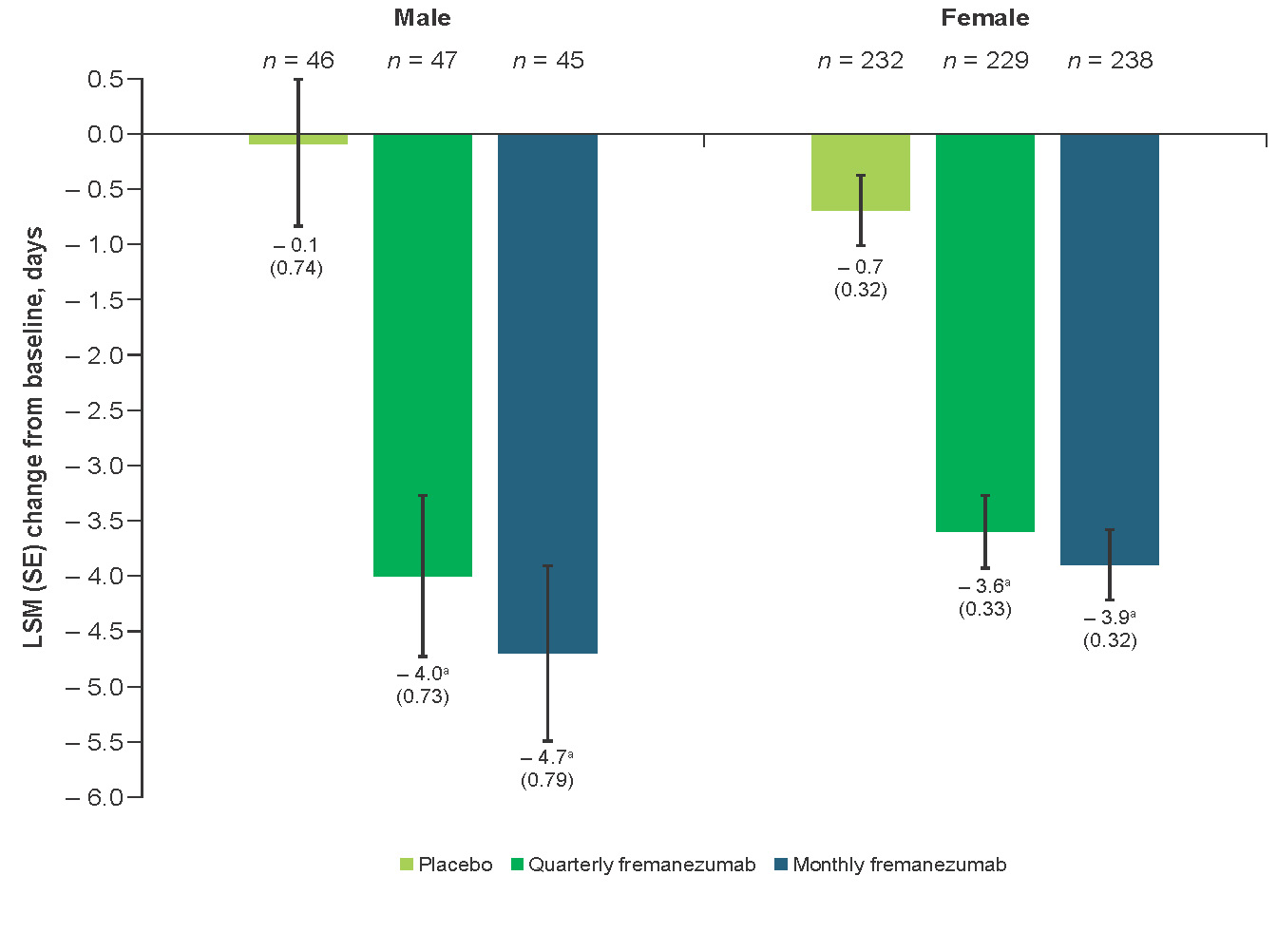
**

LSM, least-squares mean; SE, standard error.

^a^*P* <0.001 vs placebo.

**Supplementary Fig. 6.** Change in HIT-6 scores at 12 weeks by sex.


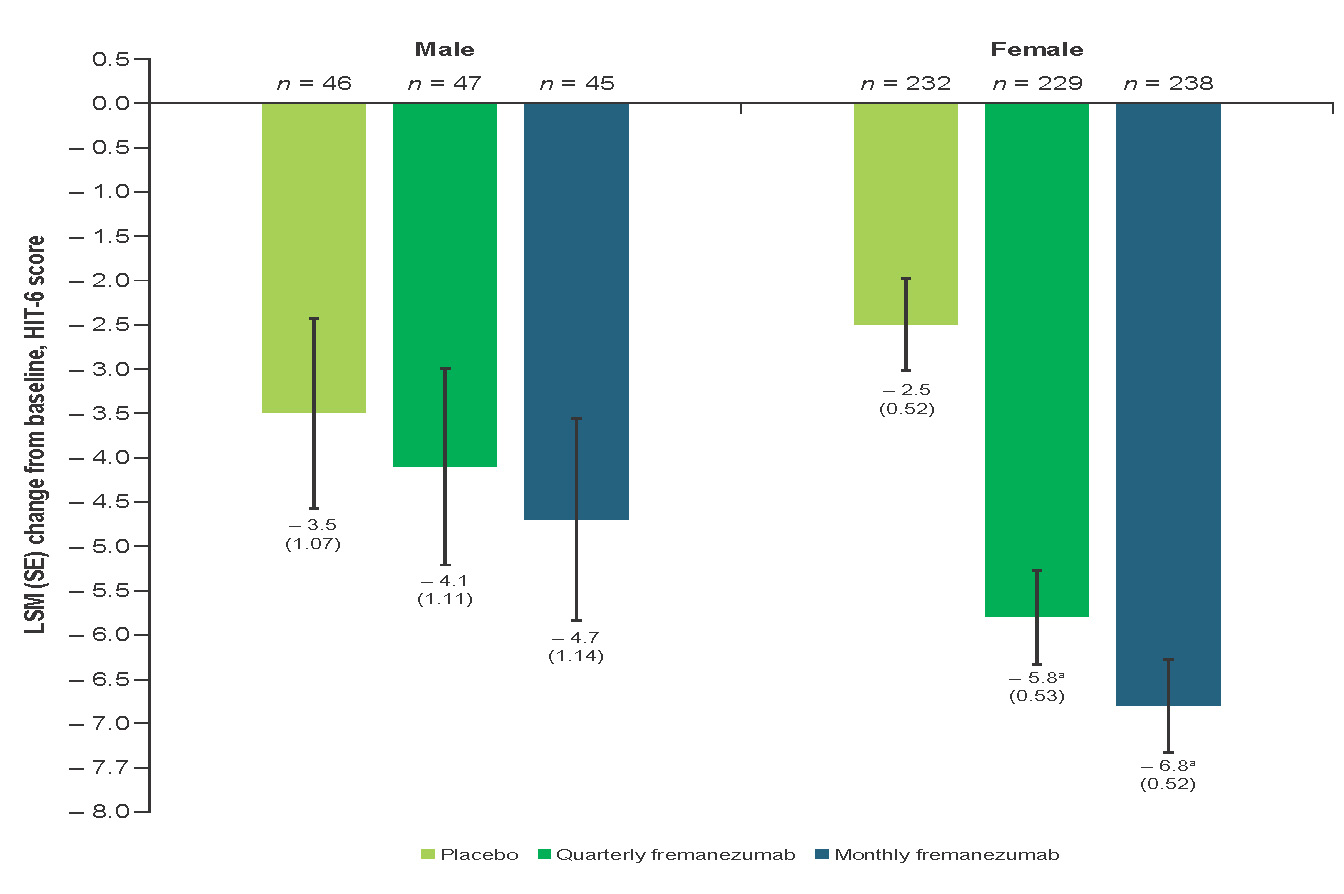


HIT-6, Headache Impact Test-6; LSM, least-squares mean; SE, standard error.

^a^*P* <0.001 vs placebo.
